# Racial inequalities in the development of multimorbidity of chronic conditions: results from a Brazilian prospective cohort

**DOI:** 10.1186/s12939-024-02201-8

**Published:** 2024-06-12

**Authors:** Fernanda Esthefane Garrides Oliveira, Rosane Härter Griep, Dora Chor, Sandhi Maria Barreto, Maria del Carmen Bisi Molina, Luciana A. C. Machado, Maria de Jesus Mendes da Fonseca, Leonardo Soares Bastos

**Affiliations:** 1 Sérgio Arouca National School of Public Health, Oswaldo Cruz Foundation, 4365 Brazil Avenue, Manguinhos, Rio de Janeiro, 21040900 Brazil; 2 Laboratory of Health and Environment Education, Oswaldo Cruz Institute, Rio de Janeiro, Brazil; 3 Department of Preventive and Social Medicine, Federal University of Minas Gerais, Belo Horizonte, Brazil; 4 Federal University of Espírito Santo, Vitória, Brazil; 5 Clinical Hospital/EBSERH, Federal University of Minas Gerais, Belo Horizonte, Brazil; 6 Scientific Computing Program, Oswaldo Cruz Foundation, Rio de Janeiro, Brazil; 7 Executive Office, Science Integrity Alliance, Sunrise, Florida US

**Keywords:** Aging, Health inequities, Health inequality monitoring, Longitudinal studies, Multimorbidity, Noncommunicable diseases, Racial inequalities in health, Racism, Social determinants of health

## Abstract

**Background:**

The occurrence of multimorbidity and its impacts have differentially affected population subgroups. Evidence on its incidence has mainly come from high-income regions, with limited exploration of racial disparities. This study investigated the association between racial groups and the development of multimorbidity and chronic conditions in the Brazilian Longitudinal Study of Adult Health (ELSA-Brasil).

**Methods:**

Data from self-reported white, brown (*pardos* or mixed-race), and black participants at baseline of ELSA-Brasil (2008–2010) who were at risk for multimorbidity were analysed. The development of chronic conditions was assessed through in-person visits and self-reported diagnosis via telephone until the third follow-up visit (2017–2019). Multimorbidity was defined when, at the follow-up visit, the participant had two or more morbidities. Cumulative incidences, incidence rates, and adjusted incidence rate ratios (IRRs) were estimated using Poisson models.

**Results:**

Over an 8.3-year follow-up, compared to white participants: browns had a 27% greater incidence of hypertension and obesity; and blacks had a 62% and 45% greater incidence, respectively. Blacks also had 58% more diabetes. The cancer incidence was greater among whites. Multimorbidity affected 41% of the participants, with a crude incidence rate of 57.5 cases per 1000 person-years (ranging from 56.3 for whites to 63.9 for blacks). Adjusted estimates showed a 20% higher incidence of multimorbidity in black participants compared to white participants (IRR: 1.20; 95% CI: 1.05–1.38).

**Conclusions:**

Significant racial disparities in the risk of chronic conditions and multimorbidity were observed. Many associations revealed a gradient increase in illness risk according to darker skin tones. Addressing fundamental causes such as racism and racial discrimination, alongside considering social determinants of health, is vital for comprehensive multimorbidity care. Intersectoral, equitable policies are essential for ensuring health rights for historically marginalized groups.

**Supplementary Information:**

The online version contains supplementary material available at 10.1186/s12939-024-02201-8.

## Additional non-English language abstract (Portuguese)

**Antecedentes e objetivos:**

A ocorrência de multimorbidade e seus impactos têm afetado de forma diferenciada os subgrupos populacionais. As evidências sobre sua incidência provêm principalmente de regiões de alta renda, com exploração limitada das disparidades raciais. Este estudo investigou a associação entre grupos raciais e o desenvolvimento de multimorbidade e de condições crônicas no Estudo Longitudinal de Saúde do Adulto (ELSA-Brasil).

**Métodos:**

Foram analisados dados de participantes autodeclarados brancos, pardos (raças mistas) e pretos na linha de base do ELSA-Brasil (2008-2010) que estavam em risco para a multimorbidade. O desenvolvimento de condições crônicas foi avaliado a partir das visitas presenciais e autorrelato de diagnóstico por telefone até a terceira visita de seguimento (2017-2019). A multimorbidade foi definida quando na visita de seguimento o participante possuía duas ou mais morbidades. Foram estimadas incidências acumuladas, taxas de incidência e razões das taxas de incidência (RTI) ajustadas a partir de modelos de Poisson.

**Resultados:**

Em um acompanhamento de 8,3 anos, em comparação aos participantes brancos: os pardos tiveram uma incidência 27% maior de hipertensão e obesidade; e os pretos tiveram uma incidência 62% e 45% maior, respectivamente. Os pretos também tiveram 58% mais diabetes. A incidência de câncer foi maior entre os brancos. A multimorbidade afetou 41% dos participantes, com uma taxa bruta de incidência de 57,5 casos por 1.000 pessoas-ano (variando de 56,3 para brancos a 63,9 para pretos). Estimativas ajustadas mostraram incidência 20% maior de multimorbidade em pretos comparados aos brancos (RTI: 1,20; IC 95%: 1,05-1,38).

**Conclusões:**

Foram observadas disparidades raciais significativas no risco de condições crônicas e multimorbidade. Muitas associações revelaram um aumento gradiente no risco de doenças de acordo com tons de pele mais escuros. Abordar causas fundamentais como o racismo e a discriminação racial, juntamente com a consideração dos determinantes sociais da saúde, é vital para o cuidado abrangente da multimorbidade. Políticas intersetoriais e equitativas são essenciais para a garantia do direito à saúde aos grupos historicamente marginalizados.

## Background

The presence of multimorbidity, defined as an individual living with at least two chronic conditions concurrently [1], is increasingly common in a scenario in which populations around the world are ageing at a faster rate and survival from illness due to acute and chronic conditions has increased [2, 3]. Nevertheless, living with multimorbidity is related to a decline in functional capacity, quality of life, and greater use of health services, both in terms of the number of visits and days spent in a hospital, higher healthcare costs, and greater risk of death [4–8]. In addition, multimorbidity adversely affects employment, leading to a worsening of absenteeism rates and temporary and permanent sick leave and influencing the employability of individuals with multimorbidity [9].

Evidence on the pathophysiology and mechanisms of multimorbidity suggests the involvement of multifactorial pathways that include various elements, including genomic, immunological, neuroendocrine, social and environmental factors [2, 10]. Mechanisms related to ageing and inflammation include, for example, cellular senescence, long-term epigenetic changes, and telomere attrition [2]. Mechanisms related to the social determinants of health range from proximal factors – such as health risk behaviours – to more distal factors, such as political and economic contexts that contribute to the vulnerability of certain population groups [11].

Amongst the proximal factors of individuals associated with multimorbidity are advancing age, although younger adults can also experience multimorbidity, and female sex [12–15]. Concerning socioeconomic determinants, lower education, lower family income, and greater socioeconomic deprivation in the area where people live have been associated with a greater prevalence of multimorbidity [15–17].

Other social determinants associated with the development of multimorbidity include race and ethnicity [18–21]. By understanding the situation of multimorbidity as the outcome of a complex response of adaptive systems to biological, behavioural and socioenvironmental factors, including stressful life experiences that promote physiological instability [10], it is possible to assume that, just as socially disadvantaged racial populations have worse outcomes for various health indicators, some racial groups will also be at greater risk of developing multimorbidity. In the same way that racial inequalities in other health indicators are observed, due to the incremental effects of racism and experiences of discrimination which, in addition to stressors, place some groups in aggravated environmental, occupational, socioeconomic positions, lacking adequate opportunity structure, material and psychological resources throughout their life course [11, 22], the hypothesis is that health inequalities are reproduced in multimorbidity.

Evidence suggests that, in the United States of America (USA), compared to white individuals, black individuals have a greater chance of multimorbidity, a greater number of coexisting diseases, and multimorbidity at a younger age [18–20]. In Brazil, previous findings have revealed a greater prevalence of multimorbidity among brown (*pardos*) and black people than among white people at baseline in a multicentre, multiracial cohort of workers [21].

Brazil is a middle-income economy and was one of the largest recipients of enslaved people in the Americas, while being one of the last countries to abolish slavery. Socioeconomic and political measures that could have provided freed enslaved people with equitable conditions for inclusion in production processes, power, and decision-making spaces were not promoted [23, 24]. Consequently, subsequent generations of these groups were historically discriminated against and marked by inequalities in various social and health indicators over the life course, culminating in lower life expectancy [25–27].

In the present study, we investigated the association between racial groups and the development of multimorbidity during an average follow-up of 8.29 years in the Brazilian Longitudinal Study of Adult Health (*Estudo Longitudinal de Saúde do Adulto* – ELSA-Brasil). Additionally, we analysed racial differences in the occurrence of morbidities throughout the follow-up period.

## Methods

### Data source and study population

The ELSA-Brasil is a multicentre prospective cohort study designed to investigate the development and progression of chronic diseases and related factors [28]. The baseline cohort, Wave 1 (2008–2010), included 15,105 civil servants aged between 35 and 74 years old from six higher education and/or research institutions located in state capital cities across three regions of Brazil: Southeast (Belo Horizonte, Rio de Janeiro, São Paulo, and Vitória), South (Porto Alegre), and Northeast (Salvador) [28]. The second visit to an ELSA facility (Wave 2) occurred between 2012 and 2014, and the third visit (Wave 3) occurred between 2017 and 2019. During in-person visits, interviews were conducted using pretested questionnaires, and clinical, laboratory and imaging tests were carried out. Additionally, participants underwent the annual follow-up interview (AFI) via phone calls to monitor their health status. Comprehensive methodological details and cohort profile can be found in previous publications [28–31].

To assess the development of multimorbidity, among the 15,105 participants in the baseline cohort, participants with missing data for any of the chronic conditions at baseline (*n* = 291) or for race/skin colour (*n* = 184) were excluded from this study. Participants self-declared as indigenous (*n* = 157) or of Asian descent (*n* = 374) were excluded due to the low frequency and infeasibility of grouping. Self-declared white, brown (*pardo*), and black participants who lived with multimorbidity at the baseline cohort were also excluded (*n* = 9882) [21].

Among the 4217 participants at risk of developing multimorbidity at the beginning of follow-up, three participants were lost during follow-up since no recorded information regarding Waves 2 and 3, the AFI or death was found. For risk assessment between Waves 1–2 and Waves 2–3, 4214 and 3091 participants were considered, respectively. In the latter, those who reached multimorbidity status up until Wave 2 (*n* = 1082), those who died up until Wave 2 (*n* = 21), and those with no information after Wave 2 (*n* = 20) were excluded. The exclusion flowchart is presented in Additional File 1.

### Outcome: new cases of multimorbidity

Multimorbidity was defined when the morbidity count at the time of Wave 2 or Wave 3 was ≥ 2, recovering the baseline information, the self-reported diagnosis of morbidities on the AFI in the period between waves, and the morbidities assessed during the in-person visits at Waves 2 and 3.

The identification of new cases of multimorbidity was established based on nine chronic morbidities that constituted the criteria for assessing multimorbidity status at baseline/Wave 1 [21]. Additional File 2 provides more details on the collection of each morbidity. Five of them were investigated during follow-up through self-reported diagnoses in the AFI, commencing with the following query: *After the last ELSA telephone interview (or last in-person visit), did a doctor tell you that you have/had (…)?* These morbidities include cancer, ischaemic heart disease (angina and/or myocardial infarction), cardiac insufficiency, cerebrovascular accident (CVA), and renal insufficiency.

Two morbidities, diabetes and hypertension, were investigated through self-reported diagnosis on the AFI and assessments during in-person visits. Diabetes was defined by self-reported diagnosis and/or medication use and/or laboratory data for fasting glycaemia (≥ 126 mg/dL), glycated haemoglobin (≥ 6.5%) and 75 g glucose tolerance test after 2 h (≥ 200 mg/dL). Hypertension was defined by self-reported diagnosis and/or systolic blood pressure (≥ 140 mmHg) and/or diastolic blood pressure (≥ 90 mmHg) and/or use of antihypertensive medication. Blood pressure estimates were based on the average of the last two measurements in a series of three taken one minute apart [31].

Two morbidities, dyslipidaemia and obesity, were exclusively investigated during in-person visits. Dyslipidaemia was defined by low-density lipoprotein cholesterol levels after a 12-hour fast (≥ 130 mg/dL) and/or the use of lipid-lowering agents. Obesity was defined by body mass index (≥ 30 kg/m²), determined through anthropometric measurements in accordance with ELSA protocols [31].

Aligned with the conceptual framework of multimorbidity, underscoring the chronic and persistent nature of morbidities accumulation and the ensuing necessity for continual care management [32], our analyses, consistent with another study [19], presumed that once an ELSA participant developed a morbidity, it persisted, even if its manifestations and symptoms were alleviated by medication and/or behavioural changes.

#### Social marker of racism: self-reported race/skin colour

Self-declared race/skin colour at the ELSA baseline was used as an independent variable in the analyses, recognized as a social construct that could unveil discriminatory processes and material stratification, serving as a proxy for exposure to systemic racism [33]. Racial self-declaration is based on the options provided by Brazil’s Official Bureau of Statistics (IBGE – *Instituto Brasileiro de Geografia e Estatística*) with the following question: *The Brazilian population census uses the terms black, pardo, white, yellow (Asian descent) and indigenous to classify people by colour or race. If you had to respond to the census today, how would you classify yourself by colour or race?* We compared browns (*pardo*) and blacks relative to whites, guided by the lower prevalence of multimorbidity among whites at baseline [21].

#### Covariates

The main confounding factors included in the analyses were age, sex, and the ELSA research centre. Inclusion of the latter was prompted by differences in (1) the racial composition of each city – Porto Alegre features a predominantly white self-reported population (79.23%), whereas Salvador’s population is predominantly brown/black (79.47%) [34]. These disparities may influence experiences of racial discrimination and the perception of these experiences. (2) The age structure of each city – Porto Alegre stands out as the city with the oldest population [34], potentially shaping local health policies targeting chronic disease prevention in an aging demographic. (3) Socioeconomic indicators and living conditions of the population differ between cities [35], which may have an impact on the devices and resources available for health promotion and disease prevention, as well as overall population health.

Socioeconomic position factors and health risk behaviours were considered for participant description and in sensitivity analyses only, as they were classified as mediators. This classification was informed by discussions and the recognition that these covariates are part of the pathway connecting race/skin colour and illness due to the effects of racism and experiences of racial discrimination [11, 22, 36, 37].

Educational attainment was categorised as complete higher education, complete high school, complete elementary school, and incomplete elementary school. Monthly *per capita* family income was categorised into quintiles based on information about monthly family income and the number of dependents on this income, thus, the first quintile corresponds to ≤ BRL 622.42 (about ≤ USD 311.58 by the 2009 average exchange rate) and the fifth quintile corresponds to > BRL 2628.17 and ≤ BRL 7884.50 (about > USD 1315.66 and ≤ USD 3946.99 by the 2009 average exchange rate). Smoking habits were categorised as nonsmoker, former smoker, and current smoker. Leisure-time physical activity was categorised as low (< 600 min/week), moderate (600–3000 min/week), and vigorous (≥ 3000 min/week), assessed using the long version of the IPAQ – International Physical Activity Questionnaire [38]. All covariates were assessed at the cohort’s baseline.

### Statistical analysis

The population at risk of developing multimorbidity at the start of follow-up was described by racial group, presenting absolute frequencies and proportions with Pearson’s chi-square test for categorical variables, and measures of position and dispersion with the Kruskal-Wallis test for age.

The times at risk for multimorbidity were defined as intervals between the dates of the visits at Wave 1 and Wave 2, between the dates of the visits at Wave 2 and Wave 3, and between the dates of the visits at Wave 1 and the participant’s longest information recording date (for the entire period between Waves 1 and 3). Participants who passed away had their time censored on the date of death, and participants identified with multimorbidity at the Wave 2 visit had their time at risk censored at Wave 2. For specifics regarding the dates considered to estimate the time at risk for each follow-up situation, please refer to the Additional File 3.

The cumulative incidence of multimorbidity was calculated by dividing the number of new multimorbidity cases by the total number of participants at risk at the beginning of the period. The incidence rate was determined by dividing the number of new multimorbidity cases by the total number of person-years at risk. For each morbidity included in the list assessed for multimorbidity, we present the cumulative incidence, estimated in a similar way to that of multimorbidity.

The relationships between racial groups and the development of each morbidity and multimorbidity were analysed through generalised linear models utilising the Poisson distribution family, logarithmic link function, and total person-time at risk in the offset. Incidence rate ratios (IRRs) and their respective 95% confidence intervals (95% CIs) are presented for models with unadjusted estimates and after adjusting for key confounders (age, sex, and research centre). All analyses were conducted using R statistical software version 4.0.3. The epiR package [39] was used to estimate the cumulative incidence and incidence rate.

Additionally, sensitivity analyses were performed to assess the association between racial groups and multimorbidity development (Additional File 4). These analyses considered the following: (1) additional adjustments for potential mediators such as socioeconomic position and health risk behaviours. (2) Outcomes focused solely on four morbidities assessed during in-person visits: hypertension, diabetes, dyslipidaemia, and obesity.

## Results

Among the 4214 self-declared white (57.1%), brown (28.7%) and black (14.2%) participants free of multimorbidity at baseline, 1338 had no morbidity at the beginning of the follow-up (31.8%), while 2876 had morbidity (68.2%), with no significant differences observed between the racial groups (Table 1). The median age was 48 years, and the white group comprised a greater proportion of participants aged > 60 years (15.2% for whites, 9.3% for browns and 10.9% for blacks). The majority of participants were male (52.5%), but females were slightly more common in the white group (50.3%). The São Paulo Research Centre accounted for the majority of participants (35.8%), of whom more than 60% (990 participants) were white, while Salvador concentrated more than 70% of brown and black participants (400 out of 514). Participants with complete higher education (56.5%), in the highest quintiles of monthly *per capita* family income (61.5% between the 3rd and 5th quintiles), nonsmokers (62.2%), and with low leisure-time physical activity (72.8%) were more prevalent. A larger proportion of whites had the highest level of education (69,4%) and were in the top two quintiles of income (52.5%), while 18.2% of black people had the lowest levels of education, and 64.8% were in the lowest income quintiles (Table 1).

**Table 1:** Descriptive characteristics of participants who were free of multimorbidity at baseline/Wave 1 (2008–2010), ELSA-Brasil

Characteristics at baseline/ Wave 1^(a)^	Total (%)	Self-declared race/skin colour group^(b)^			*p*-value^(c)^
		White (%)	Brown (%)	Black (%)	
Participants	4214 (100)	2406 (57.1)	1210 (28.7)	598 (14.2)	
Morbidities at baseline					
none	1338 (31.8)	732 (30.4)	414 (34.2)	192 (32.1)	0.068
one	2876 (68.2)	1674 (69.6)	796 (65.8)	406 (67.9)	
Age, in years					
minimum - maximum	35–75	35–75	35–74	35–74	< 0.001
mean (standard deviation)	49.17 (8.5)	49.64 (8.8)	48.39 (8.0)	48.87 (8.0)	
median (1st quartile − 3rd quartile)	48 (43–55)	48 (43–56)	47 (43–54)	48 (43–55)	
Age groups					
up to 39 years	522 (12.4)	295 (12.3)	162 (13.4)	65 (10.9)	0.002
40–44 years	875 (20.8)	482 (20.0)	263 (21.7)	130 (21.7)	
45–49 years	994 (23.6)	543 (22.6)	296 (24.5)	155 (25.9)	
50–54 years	701 (16.6)	384 (15.9)	219 (18.1)	98 (16.4)	
55–59 years	580 (13.8)	337 (14.0)	158 (13.1)	85 (14.2)	
60–64 years	317 (7.5)	212 (8.8)	64 (5.3)	41 (6.9)	
65–69 years	149 (3.5)	100 (4.2)	32 (2.6)	17 (2.8)	
70 years or more	76 (1.8)	53 (2.2)	16 (1.3)	7 (1.2)	
Sex					
male	2211 (52.5)	1196 (49.7)	709 (58.6)	306 (51.2)	< 0.001
female	2003 (47.5)	1210 (50.3)	501 (41.4)	292 (48.8)	
Research Centre					
São Paulo	1508 (35.8)	990 (41.1)	324 (26.8)	194 (32.4)	< 0.001
Belo Horizonte	905 (21.5)	476 (19.8)	318 (26.3)	111 (18.6)	
Porto Alegre	521 (12.3)	404 (16.8)	48 (3.9)	69 (11.5)	
Salvador	514 (12.2)	114 (4.7)	252 (20.8)	148 (24.7)	
Rio de Janeiro	481 (11.4)	285 (11.9)	146 (12.1)	50 (8.4)	
Vitória	285 (6.8)	137 (5.7)	122 (10.1)	26 (4.4)	
Educational attainment					
complete higher education	2382 (56.5)	1669 (69.4)	527 (43.6)	186 (31.1)	< 0.001
complete high school	1429 (33.9)	616 (25.6)	510 (42.1)	303 (50.7)	
complete elementary school	233 (5.5)	69 (2.9)	99 (8.2)	65 (10.9)	
incomplete elementary school	170 (4.1)	52 (2.1)	74 (6.1)	44 (7.3)	
Monthly *per capita* family income					
5th (> US$ 1315.66 and ≤ US$ 3946.99)	680 (16.2)	513 (21.4)	129 (10.7)	38 (6.4)	< 0.001
4th (> US$ 882.88 and ≤ US$ 1315.66)	1030 (24.6)	744 (31.1)	210 (17.4)	76 (12.8)	
3rd (> US$ 519.25 and ≤ US$ 882.88)	870 (20.7)	530 (22.1)	245 (20.3)	95 (16.0)	
2nd (> US$ 311.58 and ≤ US$ 519.25)	836 (19.9)	359 (15.0)	306 (25.4)	171 (28.7)	
1st (≤ US$ 311.58)	781 (18.6)	250 (10.4)	316 (26.2)	215 (36.1)	
Smoking					
nonsmoker	2621 (62.2)	1502 (62.4)	747 (61.7)	372 (62.2)	0.015
former smoker	1056 (25.1)	631 (26.2)	290 (24.0)	135 (22.6)	
current smoker	537 (12.7)	273 (11.4)	173 (14.3)	91 (15.2)	
Leisure-time physical activity					
low	3017 (72.8)	1635 (69.3)	902 (75.7)	480 (81.0)	< 0.001
moderate	715 (17.2)	465 (19.7)	173 (14.5)	77 (13.0)	
vigorous	413 (10.0)	260 (11.0)	117 (9.8)	36 (6.0)	

*Notes *(a) The total number of participants with complete information for each characteristic is 4214, except for income (*n* = 4197) and physical activity (*n* = 4145). (b) Proportions within each racial group and overall (summing to 100% in the column), except for the variable age in years and the total number of participants (summing to 100% in the row). (c) Refers to the Chi-squared test for proportion difference and Kruskal-Wallis for age among racial groups

### Development of multimorbidity

The average follow-up time between Wave 1 and Wave 2 was 3.86 years (ranging from 1.05 to 6.02 years). Over this period, the total person-years at risk were 16259.41, and 1082 participants achieved multimorbidity (25.68% of the total of 4214 participants). This corresponded to a crude incidence rate of 66.55 cases of multimorbidity (95% CI: 62.64–70.63) per 1000 person-years at risk, varying from 63.70 for whites to 67.88 for browns and 75.07 for blacks (Table 2). Between Waves 2 and 3, there were 637 new cases (20.61% of the total number at risk at the end of Wave 2) of multimorbidity, representing 46.64 new cases per 1000 person-years at risk during this period (95% CI: 43.09–50.41). Considering the entire follow-up period between Wave 1 and Wave 3, the average follow-up time was 8.29 years (ranging from 3.79 to 11.06 years). Throughout this period, the total person-years at risk were 29915.36, resulting in 1719 occurrences of multimorbidity (40.79% of the total of 4214 participants at risk in the cohort baseline). This resulted in a crude incidence rate of 57.46 cases of multimorbidity (95% CI: 54.78–60.24) per 1000 person-years at risk, varying from 56.34 for whites to 56.58 for browns and 63.92 for blacks. Although there were indications of higher cumulative incidences and incidence rates for black participants, the confidence intervals between the three racial groups overlapped (Table 2).

**Table 2:** Occurrence of multimorbidity by racial group, Wave 1 (2008–2010) through Wave 3 (2017–2019), ELSA-Brasil

Follow-up Period	Total participants at risk at the beginning of the period	Deaths during the inter-wave period	New cases of multimorbidity	Total person-years at risk in the period	Cumulative incidence per 100 people at risk (95% CI)	Crude incidence rate per thousand person-years at risk (95% CI)
Between Waves 1 and 2						
Total	4214	21	1082	16259.41	25.68 (24.36–27.02)	66.55 (62.64–70.63)
White	2406	11	586	9199.28	24.36 (22.62–26.12)	63.70 (58.65–69.07)
Brown (*pardo*)	1210	6	321	4729.06	26.53 (24.06–29.11)	67.88 (60.66–75.73)
Black	598	4	175	2331.07	29.26 (25.64–33.09)	75.07 (64.36–87.06)
Between Waves 2 and 3						
Total	3091	76	637	13655.94	20.61 (19.19–22.08)	46.64 (43.09–50.41)
White	1795	31	382	7982.28	21.28 (19.41–23.25)	47.86 (43.18–52.90)
Brown (*pardo*)	878	31	164	3843.00	18.68 (16.15–21.42)	42.68 (36.39–49.73)
Black	418	14	91	1830.67	21.77 (17.91–26.04)	49.71 (40.02–61.03)
Between Waves 1 and 3						
Total	4214	97	1719	29915.36	40.79 (39.30-42.29)	57.46 (54.78–60.24)
White	2406	42	968	17181.56	40.23 (38.27–42.22)	56.34 (52.85-60.00)
Brown (*pardo*)	1210	37	485	8572.06	40.08 (37.31–42.91)	56.58 (51.66–61.85)
Black	598	18	266	4161.74	44.48 (40.45–48.57)	63.92 (56.47–72.08)

*Abbreviations* 95% CI – 95% Confidence Interval

Table 3 displays the IRRs for the associations between racial groups and the development of multimorbidity. According to the unadjusted models, no significant difference in incidence rates was observed between the racial groups. However, there was an indication that browns, particularly blacks, had a greater rate of developing multimorbidity. Upon adjusting the models, the strength of the association increased, revealing significant differences: between Waves 1 and 2, browns and blacks had 19% (IRR: 1.19; 95% CI: 1.03–1.37) and 28% (IRR: 1.28; 95% CI: 1.07–1.52) greater rates of developing multimorbidity than whites, respectively. Throughout the entire follow-up period, between Waves 1 and 3, the incidence of blacks differed from the incidence of multimorbidity for whites, being 20% greater (IRR: 1.20; 95% CI: 1.05–1.38). Meanwhile, the incidence of browns was 12% greater, with a borderline 95% CI (IRR: 1.12; 95% CI: 1.00-1.26). Notably, a gradient in which the association between the incidence of developing multimorbidity widened when the comparison category shifted from white and brown to white and black (Table 3) was also observed.

**Table 3:** Association between racial groups and multimorbidity development, Wave 1 (2008–2010) through Wave 3 (2017–2019), ELSA-Brasil

Follow-up Period^(a)^	Unadjusted IRR (CI 95%)	Adjusted IRR (CI 95%)^(b)^
Between Waves 1 and 2 (*n* = 4214)		
Brown (*pardo*)	1.07 (0.93–1.22)	1.19 (1.03–1.37)*
Black	1.18 (1.00-1.40)	1.28 (1.07–1.52)**
Between Waves 2 and 3 (*n* = 3091)		
Brown (*pardo*)	0.89 (0.74–1.07)	0.99 (0.82–1.20)
Black	1.04 (0.83–1.31)	1.07 (0.84–1.35)
Between Waves 1 and 3 (*n* = 4214)		
Brown (*pardo*)	1.00 (0.90–1.12)	1.12 (1.00-1.26)
Black	1.13 (0.99–1.30)	1.20 (1.05–1.38)**

*Abbreviations* IRR – Incidence Rate Ratio; 95% CI – 95% Confidence Interval. *Notes* (a) the reference category in all models is self-declared white race/skin colour, and the n in parentheses indicates the total number of participants at risk at the beginning of the period. (b) Model adjusted for age, sex, and ELSA research centre. Significance: *** *p*-value ≤ 0.001; ** 0.001 < *p*-value ≤ 0.01; * 0.01 < *p*-value < 0.05

### Occurrence of each morbidity

**Fig. 1:**
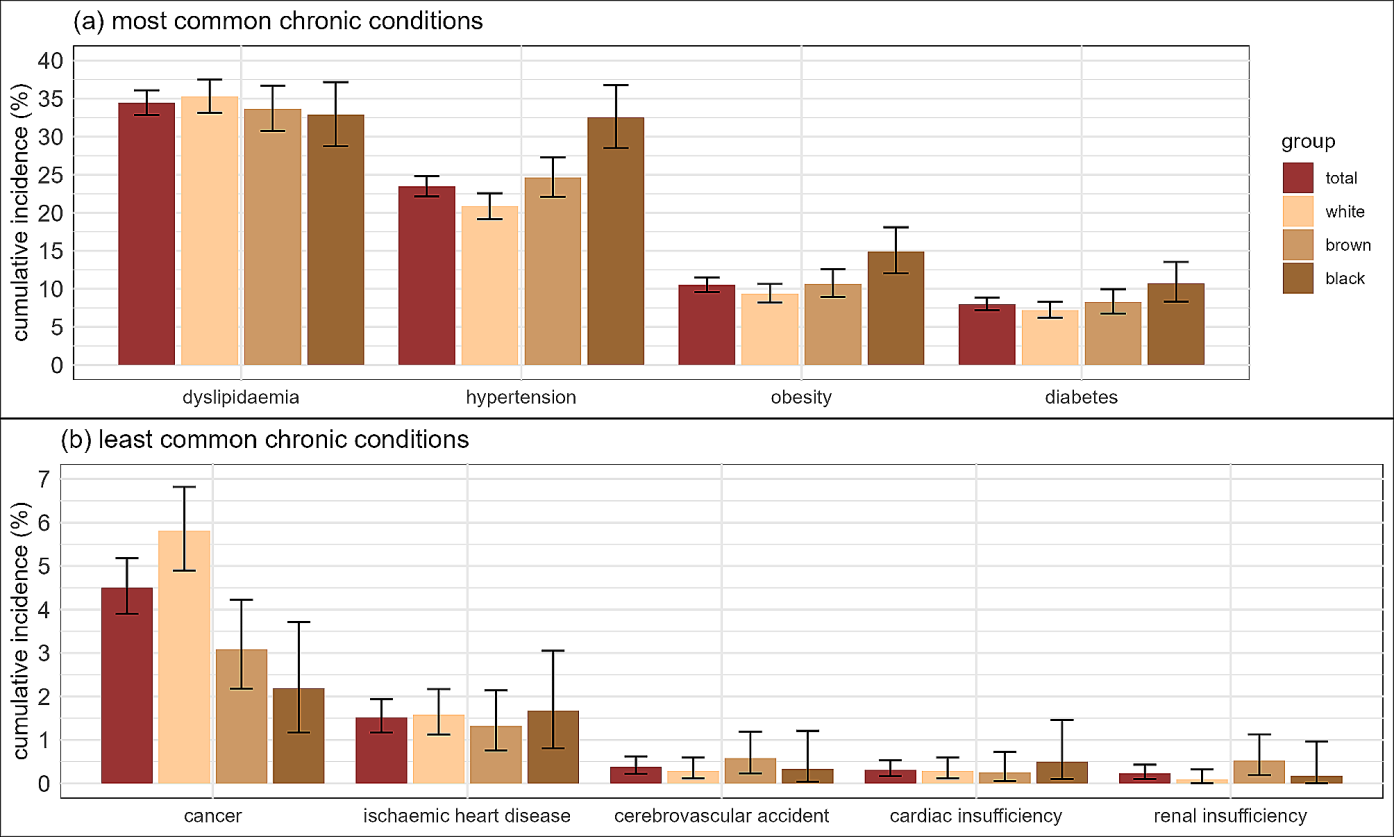
Cumulative incidence of morbidities by racial group, Wave 1 (2008–2010) through Wave 3 (2017–2019), ELSA-Brasil. *Notes* the vertical lines on the bars indicate the 95% confidence intervals

The morbidity that had the highest number of cases over the entire period was dyslipidaemia (Fig. 1 – panel a), accounting for 1154 new cases (out of a total of 3350 participants at risk at the beginning of follow-up) and representing a cumulative incidence of 34.5%. There were 906 new cases of hypertension (at risk: 3860, incidence of 23.4%), 423 cases of obesity (at risk: 4023, incidence of 10.5%), and 329 new cases of diabetes (at risk: 4119, incidence of 8%). In contrast, the morbidities that occurred least often during this period were cerebrovascular accident, cardiac insufficiency, and renal insufficiency (Fig. 1 – panel b), with 16 (0.38%), 13 (0.31%), and 9 (0.23%) new cases, respectively. Analyses of the confidence intervals for the cumulative incidences of hypertension, obesity, and diabetes by racial group revealed significant differences when comparing white and black participants. However, no significant differences were detected between white and brown or brown and black. Concerning cancer incidence, indications of differences were found between white and black participants and between white and brown participants (Fig. 1 – panel b). Further information regarding the prevalence of each morbidity in each wave and new cases in the period can be found in Additional File 5.

Table 4 shows the IRRs for the associations between racial groups and the development of each of the morbidities assessed to define multimorbidity. According to the unadjusted models, compared to those of white participants, the incidence rates of hypertension and obesity were greater for browns and blacks, in that order. Diabetes rate was greater for black participants, whereas the renal insufficiency rate was greater for brown participants, and the incidence rate of cancer was greater for white participants than for brown or black participants. After controlling for the primary confounders (adjusted IRRs), these relationships persisted, and the strength of the association increased. Browns exhibited a 27% greater incidence of hypertension (IRR: 1.27; 95% CI: 1.08–1.49) and obesity (IRR: 1.27; 95% CI: 1.08–1.50) than whites did. However, blacks had a 62% (IRR: 1.62; 95% CI: 1.35–1.95) and 45% (IRR: 1.45; 95% CI: 1.19–1.75) increase in the incidence of hypertension and obesity, respectively. In contrast, compared with white participants, brown and black participants had 42% (IRR: 0.58; 95% CI: 0.39–0.85) and 61% (IRR: 0.39; 95% CI: 0.22–0.69) lower incidence rates of cancer, respectively. Moreover, the incidence rate of diabetes was 58% greater in black participants than in white participants (IRR: 1.58; 95% CI: 1.17–2.13). After adjustment for confounding factors, the association with cerebrovascular accident became significant for brown participants (Table 4).

**Table 4:** Association between racial groups and morbidity development, Wave 1 (2008–2010) through Wave 3 (2017–2019), ELSA-Brasil

Association with each morbidity^(a)^	Unadjusted IRR (CI 95%)	Adjusted IRR (CI 95%)^(b)^
Dyslipidaemia (*n* = 3350)		
Brown (*pardo*)	0.96 (0.84–1.09)	1.04 (0.90–1.19)
Black	0.93 (0.79–1.11)	1.00 (0.84–1.18)
Hypertension (*n* = 3860)		
Brown (*pardo*)	1.18 (1.01–1.37)*	1.27 (1.08–1.49)***
Black	1.61 (1.35–1.92)***	1.62 (1.35–1.95)***
Obesity (*n* = 4029)		
Brown (*pardo*)	1.17 (1.001–1.37)*	1.27 (1.08–1.50)***
Black	1.42 (1.18–1.72)***	1.45 (1.19–1.75)***
Diabetes (*n* = 4119)		
Brown (*pardo*)	1.15 (0.90–1.47)	1.25 (0.96–1.63)
Black	1.50 (1.12–2.01)**	1.58 (1.17–2.13)***
Cancer (*n* = 4174)		
Brown (*pardo*)	0.52 (0.36–0.75)***	0.58 (0.39–0.85)**
Black	0.37 (0.21–0.66)***	0.39 (0.22–0.69)***
Ischaemic heart disease (*n* = 4207)		
Brown (*pardo*)	0.83 (0.47–1.50)	1.02 (0.55–1.90)
Black	1.06 (0.53–2.12)	1.26 (0.62–2.58)
Cerebrovascular accident (*n* = 4211)		
Brown (*pardo*)	1.99 (0.70–5.67)	3.75 (1.21–11.60)**
Black	1.15 (0.24–5.54)	1.84 (0.37–9.07)
Cardiac insufficiency (*n* = 4210)		
Brown (*pardo*)	0.85 (0.22–3.29)	1.08 (0.27–4.40)
Black	1.73 (0.45–6.68)	2.46 (0.63–9.61)
Renal insufficiency (*n* = 4005)		
Brown (*pardo*)	5.89 (1.19–29.16)*	12.68 (2.40-66.91)***
Black	1.97 (0.18–21.76)	3.07 (0.28–34.30)

*Abbreviations* IRR – Incidence Rate Ratio; 95% CI – 95% Confidence Interval. *Notes* (a) the reference category in all models is self-declared white race/skin colour, and the n in parentheses indicates the total number of participants at risk at the beginning of the period. (b) Model adjusted for age, sex, and ELSA research centre. Significance: *** *p*-value ≤ 0.001; ** 0.001 < *p*-value ≤ 0.01; * 0.01 < *p*-value < 0.05

### Sensitivity analyses

According to the sensitivity analyses (Additional File 4), the addition of socioeconomic factors and health risk behaviours to the models examining the association between racial groups and multimorbidity (see Supplementary Table 1, Additional File 4) attenuated the observed estimate in the models adjusted for age, sex, and research centre for the period between Waves 1 and 2. The estimate increased for the period between Waves 2 and 3 and remained consistent for the period between Waves 1 and 3. Nevertheless, controlling for these factors in these models was insufficient to eliminate the disparities in multimorbidity incidence rates among brown/black participants compared to white participants between Waves 1 and 2 and, similarly, among black participants compared to white participants between Waves 1 and 3.

When the outcome was altered (see Supplementary Table 1, Additional File 4), considering a list of four morbidities assessed through in-person visits involving clinical and laboratory tests (dyslipidaemia, hypertension, obesity, and diabetes), the associations remained consistent during Waves 1 and 2. The magnitude of association intensified between Waves 2 and 3, albeit without significant disparities. Additionally, across the entire period (Waves 1 to 3), the strength of the association increased, and the confidence interval for the estimate regarding brown participants was no longer marginal: brown participants exhibited a 16% greater incidence rate of multimorbidity (IRR: 1.16; 95% CI: 1.02–1.32), while black participants showed a 29% greater incidence (IRR: 1.29; 95% CI: 1.11–1.50).

## Discussion

In a multiracial and multicentre cohort of Brazilian adults and elderly individuals, we observed substantial racial disparities in both the risk of individual morbidities and the development of multimorbidity over an average follow-up period of 8.29 years. Specifically, compared to white participants, black participants exhibited a greater incidence of diabetes, and brown and black participants had elevated incidence rates of hypertension and obesity, as well as greater rates of developing multimorbidity. Moreover, for the main chronic conditions and for multimorbidity, an increase in incidence was observed according to the racial categories related to darker skin tones.

At the beginning of Wave 3 of the ELSA-Brasil study, 40.79% of the self-declared white participants, brown participants, and black participants who were initially free of multimorbidity at baseline had developed two or more morbidities. This estimate closely paralleled findings from a study conducted in the USA involving adults aged between 50 and 74 at baseline, which reported a 39.7% new case incidence rate of multimorbidity over an 8-year period. This study assessed reports of medical diagnoses of arthritis, diabetes, heart disease, hypertension, and lung disease [40]. However, our estimate differs from those in European studies, which often include younger individuals or have shorter follow-up periods. In population-based studies, a cumulative incidence of multimorbidity was 30.8% in Norway for adults aged > 20 to 59 years reassessed 11 years later based on 17 morbidities (11 by reported medical diagnosis) [41] and 30.4% in Ireland for adults aged 50 and older from the Irish Longitudinal Study on Aging reassessed two years later based on 16 morbidities/groups of morbidities [42].

Regarding the incidence rate, our study revealed 57.46 new cases of multimorbidity per 1000 person-years during the average follow-up period of 8.29 years. In the Whitehall II study involving British civil servants, analysis of a median follow-up of 23.6 years for participants aged 50 at the beginning of follow-up indicated a multimorbidity incidence rate of 13.87 per 1000 person-years. The Whitehall II study considered nine morbidities, including three common conditions in our study (diabetes, CVA and cancer), while the remaining conditions were not included in our assessment, such as Parkinson’s disease and dementia [43].

Although our definition of multimorbidity incidence aligns with the commonly used approach in the field — marking the recording of the second chronic morbidity [44] — and our study shares common morbidities with other investigations, such as diabetes, hypertension, dyslipidaemia, and obesity [41, 42], estimations can diverge due to the variety of morbidities used to define multimorbidity. European studies, for example, typically include primary causes of death in high-income countries, including neurodegenerative diseases and other age-related conditions [41, 43]. Nevertheless, our estimates indicate the potential prevalence of multimorbidity in low- and middle-income regions, suggesting that multimorbidity occurrence in the ELSA-Brasil cohort surpasses that reported in studies conducted in high-income nations. A review on the incidence of multimorbidity [44] reported a median cumulative incidence of 2.8% and a median incidence rate of 30.7 cases per 1000 person-years, estimates that mainly reflect populations in high-income regions.

There is a noticeable dearth of studies investigating multimorbidity in low- and middle-income countries [45], particularly in terms of estimating its incidence. The prevalence of multimorbidity in these regions is also as high as in high-income countries, with an estimated global prevalence of 36.4% (95% CI: 32.2-40.6%) in a meta-analysis [46], ranging from 0.7% in a population aged ≥ 20 years in a rural western India community to 81.3% in those aged ≥ 60 years in southern Brazil [46]. However, limited information is available regarding the incidence of multimorbidity in these regions. This contrasts with the adverse and challenging context experienced by low- and middle-income countries, with a rapidly ageing population, food insecurity, cities marked by social and environmental inequalities and deeply affected by climate change [45, 47]. These regions also grapple a cross-load between noncommunicable diseases, infectious diseases and external causes in contexts of economic crises [3, 48]. These factors tend to exacerbate the population’s health situation, render health systems more fragile, and escalate inequalities in disease prevention, access to diagnosis, and treatment [45].

In terms of racial disparities in multimorbidity, our ability to make comparisons is limited due to the scarcity of studies reporting incidence rates among different racial groups. However, existing evidence suggests a greater chance of multimorbidity in black adults than in white adults in both the USA [18–20] and Brazil [21]. From the risk perspective, in retrospective cohort analyses of primary care in the UK [49], black patients had a greater risk of having a zero-to-one morbidity than white patients did (HR = 1.36; 95% CI = 1.33–1.39), and also for one-to-two morbidities (HR = 1.25; 95% CI = 1.20–1.30). A study that analysed administrative data from Olmsted (Minnesota, USA), spanning 2000–2013, estimated age- and sex-standardised multimorbidity incidence rates of 48.5 cases per 1000 person-years at risk for black women; this estimate was 38.9 for black men, 39.4 for white women, and 36.0 for white men [50].

In our study, findings from the model adjusted for confounding factors indicated a 20% greater incidence of multimorbidity among blacks than among whites, which was maintained even after controlling for socioeconomic status and risk behaviours (Additional File 4). Taken together, this evidence points to the need to understand the mechanisms underlying illness and differential accumulation of diseases between racial groups throughout the life course.

In Brazil, racial inequalities are present from the beginning of life. Children born to brown and black mothers have a greater chance of having a low birth weight [25]; a greater risk of death in the first five years of life; and a greater risk of dying from malnutrition, diarrhoea, influenza and pneumonia than children born to white mothers [26]. Census data also indicate important inequalities in the context of life and health, in cities composed mainly of brown and black people, there is greater social vulnerability, income concentration, higher rates of infant mortality and premature mortality (< 65 years), and lower life expectancy [27]. A baseline study of the ELSA-Brasil cohort further indicated that brown and black individuals often reside in economically segregated neighbourhoods and that living in these areas was associated with a greater risk of hypertension and diabetes [51]. This evidence suggests that adverse experiences disproportionately impact racial groups across their life course, potentially influencing access to resources for health promotion and disease prevention, consequently influencing the likelihood of becoming ill in adulthood.

In addition, it is important to note that there are racial differences in other stressful life experiences that promote physiological instability, requiring a complex response from adaptive systems and potentially leading to multimorbidity [10]. A study of black adults in the USA taking part in the National Survey of American Life found that those who reported higher levels of discrimination, as assessed by the Everyday Discrimination Scale, were 139% more likely to have multimorbidity of physical conditions than their peers without reports of discrimination (OR: 2.39; 95% CI: 1.74–3.29), a 436% greater chance of multimorbidity of psychiatric conditions (OR: 5.36; 95% CI: 3.28–8.75), a 431% greater chance of mixed multimorbidity—at least one physical and one psychiatric (OR: 5.31; 95% CI: 3.67–7.67), and a 228% greater chance of any multimorbidity (OR: 3.28; 95% CI: 2.67–5.45) [52]. The ELSA-Brasil carried out cross-cultural adaptation of the same scale used in this study and applied it in Wave 3 [53], which allows future studies to test the hypothesis that the experience of discrimination in everyday life may be a mediator that connects race to the incidence of multimorbidity.

Our investigation also revealed racial disparities in the occurrence of individual morbidities. Brown and black participants exhibited higher incidence rates of hypertension and obesity than whites did. Additionally, brown participants had a greater incidence rate of renal insufficiency and CVA than white participants did, while black participants exhibited a greater incidence of diabetes than white participants did. These findings align with other analyses of incidence within the Brazilian context [54–56] and with what is known about how stressful experiences in the life course of the black population and worse social conditions can favour the development of these morbidities [11, 22]. It is important to mention that five causes that we analysed and indicated different occurrences among racial groups are among the ten causes that had the greatest absolute increases in the number of disability-adjusted life years lost (DALYs) in the world between 1990 and 2019 (ischaemic diseases, diabetes, CVA, kidney disease and cancer) [3]. This underscores the substantial burden of morbidities on healthcare systems and emphasises the challenge of addressing population health issues equitably, taking into account the social determinants of health.

In our study, the adjusted model revealed a significant difference in the incidence of renal insufficiency between brown and white participants but not between black and white participants. A previous baseline analysis from the ELSA-Brasil examining the albumin‒creatinine ratio and glomerular filtration rate revealed alterations in these indicators in 11.1% of black participants, 9.2% of brown participants, and 7.9% of white participants [57]. However, when considering self-reported diagnoses of kidney disease at baseline, the prevalence was highest among white participants, followed by brown and black participants [21]. This leads us to believe that black participants may be underdiagnosed in relation to renal insufficiency since they are related to hypertension and diabetes, which are more common in this population, as our findings also indicate.

Findings from Brazil’s 2019 National Health Survey revealed a prevalence of chronic kidney disease (self-reported diagnosis) of 1.6% among white individuals, 1.3% among brown individuals, and 1.2% among black individuals [58]. An analysis focused on patients receiving kidney dialysis under Brazil’s Unified Health System indicated that the largest proportion of these patients were white (45.8%), followed by brown (30.7%) and black (9.4%) [59]. Taken together, these results suggest potential challenges in diagnosis and healthcare access, particularly among black individuals, and possibly lower survival rates.

The same relationship was observed for cerebrovascular accidents: brown participants exhibited a greater incidence rate than white participants did, and while there was an indication that black participants also had a greater incidence than white participants did, the difference was not statistically significant. A meta-analysis encompassing data from 29 countries involving adults aged between 18 and 50 years who experienced a first ischaemic CVA highlighted that the black demographic presented the highest prevalence of two or more risk factors for CVA. In this group, hypertension (52.10%), diabetes (20.7%), and obesity (44.6%) were more prevalent, while dyslipidaemia was more common among white individuals (40.4%) [60]. Our study’s findings differ from expected, as we did not observe differences in CVA incidence rates between black and white participants, even though in the ELSA cohort, the incidence rates of morbidities that are risk factors for CVA were greater for black participants. One plausible explanation for this outcome might be the insufficient follow-up time for the occurrence of CVA, given that the study group included participants who were less ill at baseline, and predominantly young workers.

Although our findings contribute to the body of relatively recent research on racial inequalities in the development of multimorbidity, they should be interpreted in light of several limitations. The use of self-reported diagnoses for five conditions may have led to an underestimation of the associations. Although evidence shows good to fair agreement for several self-reported and doctor-reported chronic conditions among patients with multimorbidity [61], in Brazil, structural racism is reproduced in health services. Despite having a public and universal healthcare system, inequalities can be observed in access to services, screening and diagnosis, and disease care [62]. In this regard, our additional analyses revealed that when considering only morbidities assessed during in-person visits at the investigation centre, the strength of the association increased and became significant for browns, indicating 16% and 29% greater incidence rates of multimorbidity for browns and blacks, respectively, than for white individuals between Waves 1 and 3 (Additional File 4). This evidence highlights a potential differential misclassification error when considering self-reported morbidities. Historically discriminated against and faced more barriers to diagnosing morbidities, browns and blacks may not report illnesses that they actually have, leading the models’ estimates to distance them from a state of illness, despite being affected.

ELSA-Brasil comprises a cohort of civil servants situated in major urban centres across Brazil, excluding individuals without formal employment relationships, those with exceptionally high incomes, those with no income, or residents in regions distant from the capital cities. In addition, the group of brown and black people in Brazil is more likely to be in informal occupations or to be unoccupied [63, 64]. Consequently, we infer that racial inequalities in the development of multimorbidity are even wider than we have been able to measure.

This study has several strengths. The ELSA-Brasil comprises a prospective cohort that uses validated instruments, standardised protocols, and health evaluations conducted by trained and certified health professionals [28–31]. The annual follow-up interviews enabled the retrieval of health-related information from participants who might not have visited the research centres during in-person return periods. This approach minimises losses in the analyses and facilitates the tracking of various health outcomes, including hospitalisations and deaths. Future research should explore whether there are racial inequalities in survival between racial groups after developing multimorbidity, as well as the impact of multimorbidity status on the use of health services and daily activities.

Although differences in the baseline characteristics of the ELSA participants can be observed, as this was a cohort of workers, the participants shared similar characteristics, such as institutional ties and labour rights. Even so, we were able to observe racial inequalities in the development of isolated morbidities and multimorbidity, which is a warning about how the life course in a social context marked by unequal racial relations can impact health in adulthood and ageing.

ELSA recruited adults ranging from 35 to 75 years old at baseline who were not institutionalised, hospitalised or recruited by health services, making it a good resource for understanding the development of multimorbidity among healthy individuals making the transition from middle to old age in the context of a middle-income country. Research into multimorbidity in younger adults deserves attention because of its effect on work [9], quality of life, use of health services, catastrophic health expenditures [4, 8], health systems and society. Particularly in low- and middle-income regions, where families living with chronic morbidities spend more on healthcare and face a greater risk of catastrophic expenses and impoverishment than families unaffected by chronic morbidities, with significant reports of nonadherence to the medicines prescribed for these morbidities due to cost [65].

Multimorbidity is a significant public health concern, not only representing a substantial social challenge but also a notable economic burden, wherein the development and accumulation of morbidities and their impact are distributed unevenly, affecting certain population groups that have historically been less advantaged and are at a higher risk for various health outcomes. Additional longitudinal studies are needed to explore multifactorial pathways and progression of illness among racial groups, as well as to evaluate the patterns and severity of morbidities and consequent impacts on activities of daily living and occupation, and to determine whether racial inequalities are reproduced in other aspects of multimorbidity and survival.

## Conclusions

Multimorbidity occurs unequally among Brazilian adults and elderly individuals, with brown and black individuals at greater risk of developing the main chronic health conditions, and black individuals more frequently developing multimorbidity. As the population ages, it becomes imperative to reconsider care models that focus on managing individual chronic conditions. Instead, there should be a shift toward addressing the complex needs of patients with multiple conditions, taking into account the social determinants of health in understanding and managing multimorbidity. Equitable health promotion and disease prevention policies should be prioritised in health action planning, with strategies targeted at populations most at risk of illness. However, to ensure the right to basic and dignified living conditions for historically discriminated groups, it is crucial to address the fundamental causes of racial inequalities, including racism and racial discrimination, across all sectors of society, not solely within the realm of health.

### Electronic supplementary material

Below is the link to the electronic supplementary material.

Supplementary Material 1

Supplementary Material 2

Supplementary Material 3

Supplementary Material 4

Supplementary Material 5

## Data Availability

The datasets used and/or analysed during the current study are available from the corresponding author upon reasonable request.

## Abbreviations

AFI: Annual Follow–up Interview
CI: Confidence interval
CIS: R–Clinical Interview Schedule–Revised Version
CVA: Cerebrovascular accident
DALY: Disability–adjusted life years
ELSA: Brasil–the name of the Brazilian Longitudinal Study of Adult Health in Portuguese (*Estudo Longitudinal de Saúde do Adulto*)
HR: Hazard ratio
IBGE: Brazil’s Official Bureau of Statistics in Portuguese (*Instituto Brasileiro de Geografia e Estatística*)
IPAQ: International Physical Activity Questionnaire
IRRs: Incidence rate ratios
OR: Odds ratio
UK: United Kingdom
USA: United States of America

## References

[1] World Health Organization. Multimorbidity: Technical Series on Safer Primary Care. Geneva: World Health Organization. 2016. https://www.who.int/publications/i/item/9789241511650. Accessed 18 Dec 2023.

[2] Skou ST, Mair FS, Fortin M, Guthrie B, Nunes BP, Miranda JJ, Boyd CM, Pati S, Mtenga S, Smith SM, Multimorbidity (2022). Nat Rev Dis Primers.

[3] GBD 2019 Diseases and Injuries Collaborators (2020). Global burden of 369 diseases and injuries in 204 countries and territories, 1990–2019: a systematic analysis for the global burden of Disease Study 2019. Lancet.

[4] Zhao Y, Atun R, Oldenburg B, McPake B, Tang S, Mercer SW, Cowling TE, Sum G, Qin VM, Lee JT (2020). Physical multimorbidity, health service use, and catastrophic health expenditure by socioeconomic groups in China: an analysis of population-based panel data. Lancet Glob Health.

[5] Makovski TT, Schmitz S, Zeegers MP, Stranges S, van den Akker M (2019). Multimorbidity and quality of life: systematic literature review and meta-analysis. Ageing Res Rev.

[6] Marengoni A, Angleman S, Melis R, Mangialasche F, Karp A, Garmen A, Meinow B, Fratiglioni L (2011). Aging with multimorbidity: a systematic review of the literature. Ageing Res Rev.

[7] Xu X, Mishra GD, Jones M (2017). Evidence on multimorbidity from definition to intervention: an overview of systematic reviews. Ageing Res Rev.

[8] Nunes BP, Flores TR, Mielke GI, Thumé E, Facchini LA. Multimorbidity and mortality in older adults: a systematic review and meta-analysis. Arch Gerontol Geriatr. 2016 Nov-Dec;67:130–8. 10.1016/j.archger.2016.07.008. 10.1016/j.archger.2016.07.008 27500661

[9] Cabral GG, Dantas de Souza AC, Barbosa IR, Jerez-Roig J, Souza DLB (2019). Multimorbidity and its impact on workers: a review of Longitudinal studies. Saf Health Work.

[10] Sturmberg JP, Bennett JM, Martin CM, Picard M (2017). Multimorbidity’ as the manifestation of network disturbances. J Eval Clin Pract.

[11] Williams DR, Lawrence JA, Davis BA, Vu C (2019). Understanding how discrimination can affect health. Health Serv Res.

[12] Nguyen H, Manolova G, Daskalopoulou C, Vitoratou S, Prince M, Prina AM (2019). Prevalence of multimorbidity in community settings: a systematic review and meta-analysis of observational studies. J Comorb.

[13] King DE, Xiang J, Pilkerton CS. Multimorbidity trends in United States adults, 1988–2014. J Am Board Fam Med. 2018 Jul-Aug;31(4):503–13. 10.3122/jabfm.2018.04.180008. 10.3122/jabfm.2018.04.180008 PMC6368177 29986975

[14] Garin N, Koyanagi A, Chatterji S, Tyrovolas S, Olaya B, Leonardi M, Lara E, Koskinen S, Tobiasz-Adamczyk B, Ayuso-Mateos JL, Haro JM (2016). Global multimorbidity patterns: a cross-sectional, Population-Based, Multi-country Study. J Gerontol Biol Sci Med Sci.

[15] Violan C, Foguet-Boreu Q, Flores-Mateo G, Salisbury C, Blom J, Freitag M, Glynn L, Muth C, Valderas JM (2014). Prevalence, determinants and patterns of multimorbidity in primary care: a systematic review of observational studies. PLoS ONE.

[16] Ingram E, Ledden S, Beardon S, Gomes M, Hogarth S, McDonald H, Osborn DP, Sheringham J (2021). Household and area-level social determinants of multimorbidity: a systematic review. J Epidemiol Community Health.

[17] Pathirana TI, Jackson CA (2018). Socioeconomic status and multimorbidity: a systematic review and meta-analysis. Aust N Z J Public Health.

[18] Johnson-Lawrence V, Zajacova A, Sneed R (2017). Education, race/ethnicity, and multimorbidity among adults aged 30–64 in the National Health interview survey. SSM Popul Health.

[19] Quiñones AR, Botoseneanu A, Markwardt S, Nagel CL, Newsom JT, Dorr DA, Allore HG. Racial/ethnic differences in multimorbidity development and chronic disease accumulation for middle-aged adults. PLoS One 2019 June 17;14(6):e0218462. 10.1371/journal.pone.0218462. 10.1371/journal.pone.0218462 PMC6576751 31206556

[20] Quiñones AR, Liang J, Bennett JM, Xu X, Ye W (2011). How does the trajectory of multimorbidity vary across Black, White, and Mexican americans in middle and old age?. J Gerontol B Psychol Sci Soc Sci.

[21] Oliveira FEG, Griep RH, Chor D, Giatti L, Machado LAC, Barreto SM, da Costa Pereira A, Fonseca MJMD, Bastos LS (2022). Racial inequalities in multimorbidity: baseline of the Brazilian Longitudinal Study of Adult Health (ELSA-Brasil). BMC Public Health.

[22] Williams DR, Lawrence JA, Davis BA (2019). Racism and health: evidence and needed research. Annu Rev Public Health.

[23] Maio MC, Santos RV (2010). Raça como questão: história, ciência e identidades no brasil [online].

[24] Munanga K (2020). Rediscutindo a mestiçagem no brasil – identidade nacional versus identidade negra.

[25] Falcão IR, Ribeiro-Silva RC, de Almeida MF, Fiaccone RL, Dos S, Rocha A, Ortelan N, Silva NJ, Paixao ES, Ichihara MY, Rodrigues LC, Barreto ML (2020). Factors associated with low birth weight at term: a population-based linkage study of the 100 million Brazilian cohort. BMC Pregnancy Childbirth.

[26] Rebouças P, Goes E, Pescarini J, Ramos D, Ichihara MY, Sena S, Veiga R, Rodrigues LC, Barreto ML, Paixão ES (2022). Ethnoracial inequalities and child mortality in Brazil: a nationwide longitudinal study of 19 million newborn babies. Lancet Glob Health.

[27] Oliveira BLCA, Luiz RR (2019). Racial density and the socioeconomic, demographic and health context in Brazilian cities in 2000 and 2010. Rev Bras Epidemiol.

[28] Aquino EM, Barreto SM, Bensenor IM, Carvalho MS, Chor D, Duncan BB, Lotufo PA, Mill JG, Molina Mdel C, Mota EL, Passos VM, Schmidt MI, Szklo M (2012). Brazilian Longitudinal Study of Adult Health (ELSA-Brasil): objectives and design. Am J Epidemiol.

[29] Chor D, Alves MG, Giatti L, Cade NV, Nunes MA, Molina MC, Benseñor IM, Aquino EM, Passos V, Santos SM, Fonseca MJ, Oliveira LC (2013). Questionário do ELSA-Brasil: desafios na elaboração de instrumento multidimensional [Questionnaire development in ELSA-Brasil: challenges of a multidimensional instrument]. Rev Saude Publica.

[30] Fedeli LG, Vidigal PG, Leite CM, Castilhos CD, Pimentel RA, Maniero VC, Mill JG, Lotufo PA, Pereira AC, Bensenor IM. Logística de coleta e transporte de material biológico e organização do laboratório central no ELSA-Brasil [Logistics of collection and transportation of biological samples and the organisation of the central laboratory in the ELSA-Brasil]. Rev Saude Publica. 2013;47 Suppl 2:63–71. Portuguese. 10.1590/s0034-8910.2013047003807. 10.1590/s0034-8910.2013047003807 24346722

[31] Mill JG, Pinto K, Griep RH, Goulart A, Foppa M, Lotufo PA, Maestri MK, Ribeiro AL, Andreão RV, Dantas EM, Oliveira I, Fuchs SC, Cunha Rde S, Bensenor IM (2013). Aferições E exames clínicos realizados nos participantes do ELSA-Brasil [Medical assessments and measurements in ELSA-Brasil]. Rev Saude Publica.

[32] Goodman RA, Posner SF, Huang ES, Parekh AK, Koh HK (2013). Defining and measuring chronic conditions: imperatives for research, policy, program, and practice. Prev Chronic Dis.

[33] Lett E, Asabor E, Beltrán S, Cannon AM, Arah OA. Conceptualizing, contextualizing, and Operationalizing Race in Quantitative Health Sciences Research. Ann Fam Med. 2022 Mar-Apr;20(2):157–63. 10.1370/afm.2792. 10.1370/afm.2792 PMC8959750 35045967

[34] Instituto Brasileiro de Geografia e Estatística (IBGE). Censo Demográfico 2010. Resultado do universo – características da população e dos domicílios. SIDRA – Sistema IBGE de Recuperação Automática. https://sidra.ibge.gov.br/pesquisa/censo-demografico/demografico-2010/universo-caracteristicas-da-populacao-e-dos-domicilios. Accessed 18 Dec 2023.

[35] Programa Cidades Sustentáveis. Mapa da desigualdade entre as capitais brasileiras 2020. Programa Cidades Sustentáveis. Fundação Ford, 1st ed. 2020. https://www.cidadessustentaveis.org.br/arquivos/link/mapa-das-desigualdades.pdf. Accessed 18 Dec 2023.

[36] Williams DR, Priest N, Anderson NB (2016). Understanding associations among race, socioeconomic status, and health: patterns and prospects. Health Psychol.

[37] Krieger N, Berkman LF, Kawachi I, Glymours MM (2014). Discrimination and health inequities. Social Epidemiology.

[38] International Physical Activity Questionnaire (IPAQ). Guidelines for Data Processing and Analysis of the International Physical Activity Questionnaire (IPAQ) – Short and Long Forms. Nov. 2005. https://sites.google.com/site/theipaq/home. Accessed 18 Dec 2023.

[39] Stevenson M, Sergeant E, epiR. Tools for the Analysis of Epidemiological Data. R package version 2.0.54, 2022. https://cran.r-project.org/web/packages/epiR/index.html.

[40] Wilson-Genderson M, Heid AR, Pruchno R (2017). Onset of multiple chronic conditions and depressive symptoms: a life events perspective. Innov Aging.

[41] Tomasdottir MO, Sigurdsson JA, Petursson H, Kirkengen AL, Ivar Lund Nilsen T, Hetlevik I, Getz L (2016). Does ‘existential unease’ predict adult multimorbidity? Analytical cohort study on embodiment based on the Norwegian HUNT population. BMJ Open.

[42] Ryan A, Murphy C, Boland F, Galvin R, Smith SM (2018). What is the impact of physical activity and physical function on the development of Multimorbidity in older adults over Time? A Population-based Cohort Study. J Gerontol Biol Sci Med Sci.

[43] Dugravot A, Fayosse A, Dumurgier J, Bouillon K, Rayana TB, Schnitzler A, Kivimaki M, Sabia S, Singh-Manoux A (2020). Social inequalities in multimorbidity, frailty, disability, and transitions to mortality: a 24-year follow-up of the Whitehall II cohort study. Lancet Public Health.

[44] Kudesia P, Salimarouny B, Stanley M, Fortin M, Stewart M, Terry A, Ryan BL (2021). The incidence of multimorbidity and patterns in accumulation of chronic conditions: a systematic review. J Multimorb Comorb.

[45] Roomaney RA, Wyk BV, Wyk VP (2022). Decolonising multimorbidity? Research gaps in low and middle-income countries. Pan Afr Med J.

[46] Asogwa OA, Boateng D, Marzà-Florensa A, Peters S, Levitt N, van Olmen J, Klipstein-Grobusch K (2022). Multimorbidity of noncommunicable diseases in low-income and middle-income countries: a systematic review and meta-analysis. BMJ Open.

[47] Hartinger SM, Yglesias-González M, Blanco-Villafuerte L, Palmeiro-Silva YK, Lescano AG, Stewart-Ibarra A, Rojas-Rueda D, Melo O, Takahashi B, Buss D, Callaghan M, Chesini F, Flores EC, Gil Posse C, Gouveia N, Jankin S, Miranda-Chacon Z, Mohajeri N, Helo J, Ortiz L, Pantoja C, Salas MF, Santiago R, Sergeeva M, de Souza T, Valdés-Velásquez A, Walawender M, Romanello M (2023). The 2022 South America report of the *Lancet* countdown on health and climate change: trust the science. Now that we know, we must act. Lancet Reg Health Am.

[48] Paes-Sousa R, Rasella D, Carepa-Sousa J (2018). Política econômica E saúde pública: equilíbrio fiscal e bem-estar Da população. Saúde Debate [Internet].

[49] Bisquera A, Turner EB, Ledwaba-Chapman L, Dunbar-Rees R, Hafezparast N, Gulliford M, Durbaba S, Soley-Bori M, Fox-Rushby J, Dodhia H, Ashworth M, Wang Y (2021). Inequalities in developing multimorbidity over time: a population-based cohort study from an urban, multiethnic borough in the United Kingdom. Lancet Reg Health Eur.

[50] St Sauver JL, Boyd CM, Grossardt BR, Bobo WV, Finney Rutten LJ, Roger VL, Ebbert JO, Therneau TM, Yawn BP, Rocca WA (2015). Risk of developing multimorbidity across all ages in an historical cohort study: differences by sex and ethnicity. BMJ Open.

[51] Barber S, Diez Roux AV, Cardoso L, Santos S, Toste V, James S, Barreto S, Schmidt M, Giatti L, Chor D (2018). At the intersection of place, race, and health in Brazil: residential segregation and cardio-metabolic risk factors in the Brazilian Longitudinal Study of Adult Health (ELSA-Brasil). Soc Sci Med.

[52] Oh H, Glass J, Narita Z, Koyanagi A, Sinha S, Jacob L (2021). Discrimination and Multimorbidity among Black americans: findings from the National Survey of American Life. J Racial Ethn Health Disparities.

[53] Griep RH, Oliveira FEG, Aguiar OB, Moreno AB, Alves MGM, Patrão AL, Fonseca MJMD, Chor D (2023). Cross-cultural adaptation of discrimination and vigilance scales in ELSA-Brasil. Rev Saude Publica.

[54] Machado AV, Camelo LV, Chor D, Griep RH, Guimarães JMN, Giatti L, Barreto SM (2021). Racial inequality, racial discrimination and obesity incidence in adults from the ELSA-Brasil cohort. J Epidemiol Community Health.

[55] da Silva EKP, Barreto SM, Brant LCC, Camelo LV, Araújo EM, Griep RH, Fonseca MJMD, Pereira ADC, Giatti L (2023). Gender, race/skin colour and incidence of hypertension in ELSA-Brasil: an intersectional approach. Ethn Health.

[56] Smolen JR, de Araújo EM, de Oliveira NF, de Araújo TM (2018). Intersectionality of race, gender, and Common Mental disorders in Northeastern Brazil. Ethn Dis.

[57] Barreto SM, Ladeira RM, Duncan BB, Schmidt MI, Lopes AA, Benseñor IM, Chor D, Griep RH, Vidigal PG, Ribeiro AL, Lotufo PA, Mill JG (2016). Chronic kidney disease among adult participants of the ELSA-Brasil cohort: association with race and socioeconomic position. J Epidemiol Community Health.

[58] Gouvêa ECDP, Szwarcwald CL, Damacena GN, Moura L. Autorrelato de diagnóstico médico de doença renal crônica: prevalência e características na população adulta brasileira, Pesquisa Nacional de Saúde 2013 e 2019. Epidemiologia e Serviços de Saúde [online]. 2022, 31(spe1): e2021385. 10.1590/SS2237-9622202200017. 10.1590/SS2237-9622202200017.especial PMC9897824 35920461

[59] de Moura L, Prestes IV, Duncan BB, Thome FS, Schmidt MI (2014). Dialysis for end stage renal disease financed through the Brazilian National Health System, 2000 to 2012. BMC Nephrol.

[60] Jacob MA, Ekker MS, Allach Y, Cai M, Aarnio K, Arauz A (2022). Global differences in risk factors, etiology, and outcome of ischemic stroke in young Adults-A Worldwide Meta-analysis: the GOAL Initiative. Neurology.

[61] Hansen H, Schäfer I, Schön G, Riedel-Heller S, Gensichen J, Weyerer S, Petersen JJ, König HH, Bickel H, Fuchs A, Höfels S, Wiese B, Wegscheider K, van den Bussche H, Scherer M (2014). Agreement between self-reported and general practitioner-reported chronic conditions among multimorbid patients in primary care - results of the MultiCare Cohort Study. BMC Fam Pract.

[62] Anunciação D, Pereira LL, Silva HP, Nunes APN, Soares JO (2022). Ways and detours in guarantee of health for the black population and the confrontation of racism in Brazil. Cien Saude Colet.

[63] Instituto Brasileiro de Geografia e Estatística (IBGE). Coordenação de Trabalho e Rendimento. Pesquisa Nacional por Amostra de Domicílios: síntese de indicadores, 2015. Rio de Janeiro: IBGE. 2016. https://biblioteca.ibge.gov.br/biblioteca-catalogo?id=298887&view=detalhes. Accessed 18 Dec 2023.

[64] Instituto Brasileiro de Geografia e Estatística (IBGE). Coordenação de População e Indicadores Sociais. Síntese de indicadores sociais: uma análise das condições de vida da população brasileira. Rio de Janeiro: IBGE. 2020. https://biblioteca.ibge.gov.br/index.php/biblioteca-catalogo?view=detalhes&id=2101979. Accessed 18 Dec 2023.

[65] Murphy A, Palafox B, Walli-Attaei M, Powell-Jackson T, Rangarajan S, Alhabib KF (2020). The household economic burden of noncommunicable diseases in 18 countries. BMJ Glob Health.

