# Supplementary material for: Racial inequalities in the development of multimorbidity of chronic conditions: results from a Brazilian prospective cohort
Source: Int J Equity Health. 2024 Jun 12;23:120. doi: 10.1186/s12939-024-02201-8 (PMC11170781; doi:10.1186/s12939-024-02201-8)

Additional File 1

Exclusion flowchart to assess the association between race/skin colour and multimorbidity development, ELSA-Brasil

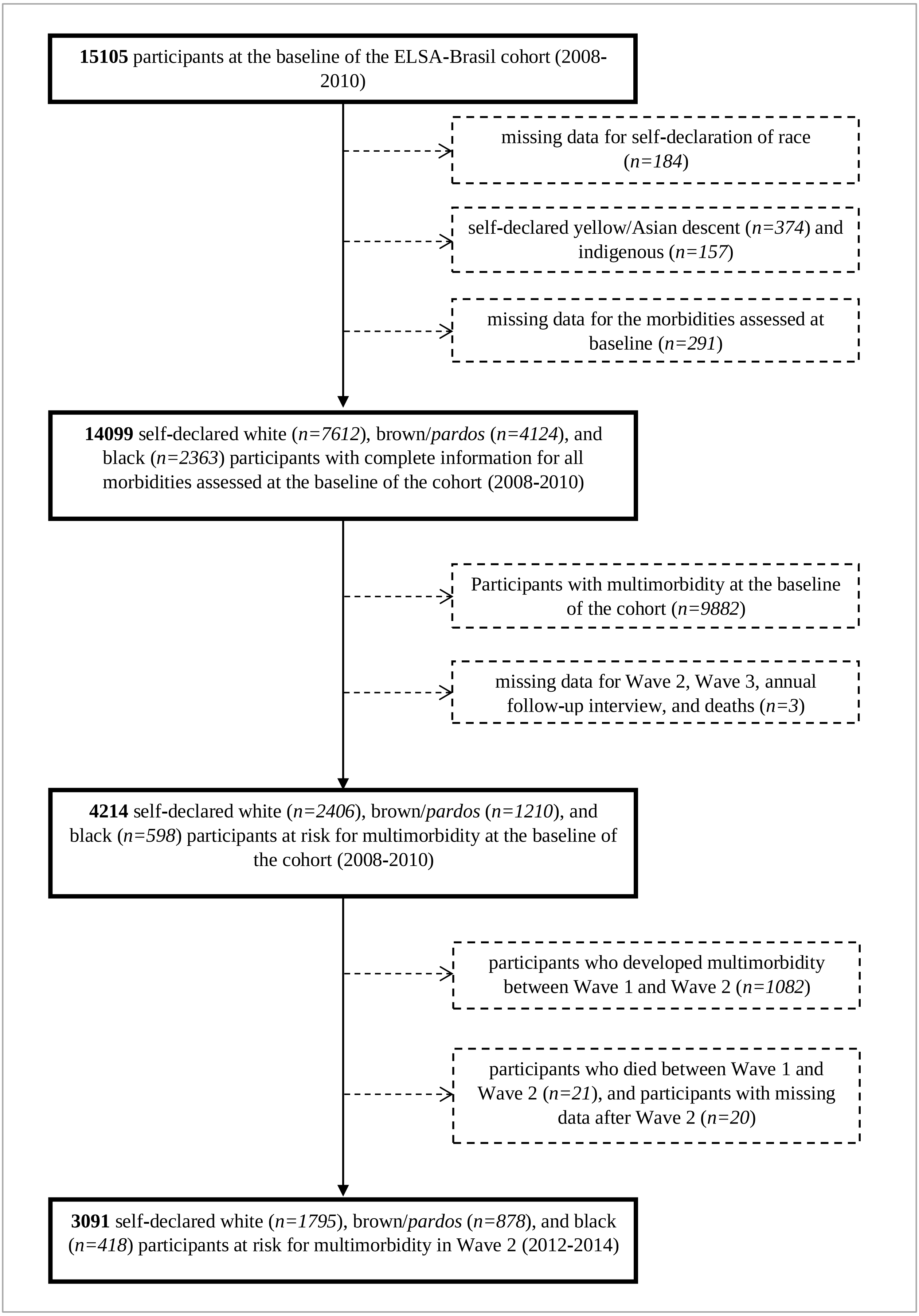

Supplement: Supplementary file 1 — Supplementary Material 1 [file 12939_2024_2201_MOESM1_ESM.pdf]
