# Supplementary material for: Racial inequalities in the development of multimorbidity of chronic conditions: results from a Brazilian prospective cohort
Source: Int J Equity Health. 2024 Jun 12;23:120. doi: 10.1186/s12939-024-02201-8 (PMC11170781; doi:10.1186/s12939-024-02201-8)
Supplement: Supplementary file 2 — Supplementary Material 2 [file 12939_2024_2201_MOESM2_ESM.pdf]

Additional File 2

Definition of the presence of each morbidity assessed in the Annual Follow-up Interview, during the in-person visit at Wave 2 (2012-2014) and Wave 3 (2017-2019) of the Brazilian Longitudinal Study of Adult Health (ELSA-Brasil)

**Morbidity**	**Assessment during the Annual Follow-up Interview**	**Assessment during the visit to Wave 2**	**Assessment during the visit to Wave 3**
cancer	After the last ELSA telephone interview (or last in-person visit), did a doctor tell you that you have/had a cancer?	-	-
cardiac insufficiency	After the last ELSA telephone interview (or last in-person visit), did a doctor tell you that you have/had cardiac insufficiency (enlarged or dilated heart)?	-	-
cerebrovascular accident	After the last ELSA telephone interview (or last in-person visit), did a doctor tell you that you have/had a cerebrovascular accident?	-	-
common nonpsychotic mental disorders	-	-	Present when the total score of the Clinical Interview Schedule – Revised Version (CIS-R) ≥12. The CIS-R is an instrument with 15 sections that assess the existence and intensity of some symptoms, duration, frequency, and time of occurrence. Symptoms evaluated in relation to the seven days preceding the participants' in-person visit included somatic symptoms; fatigue; problems with concentration and memory; sleep problems; irritability; concerns about physical health; feeling sad or depressed; depressive ideas; worries; feeling anxious or nervous; phobias; panic; compulsions; and obsessions. The last section assesses the overall effects of symptoms on the interviewee's quality of life.

Definition of the presence of each morbidity assessed in the Annual Follow-up Interview, during the in-person visit at Wave 2 (2012-2014) and Wave 3 (2017-2019) of the Brazilian Longitudinal Study of Adult Health (ELSA-Brasil)

**Morbidity**	**Assessment during the Annual Follow-up Interview**	**Assessment during the visit to Wave 2**	**Assessment during the visit to Wave 3**
diabetes	After the last ELSA telephone interview (or last in-person visit), did a doctor tell you that you have diabetes (mellitus)?	Self-report of diagnosis; and/or medication use; and/or through laboratory data for glycated haemoglobin equal to or greater than 6.5%, assessed by high-pressure liquid chromatography (Bio-Rad Laboratories, Hercules, California), using a method certified by the National Glycohemoglobin Standardization Program; and/or fasting glucose levels equal to or greater than 126mg/dL; and/or glucose tolerance test after ingestion of 75 grams of dextrose (anhydrous glucose) equal to or greater than 200mg/dL of glucose after 2 hours, conducted in participants without known diabetes. In those with diabetes, the oral glucose intake was replaced by a standardized food load. Fasting glucose and glucose tolerance test were evaluated using the hexokinase method (enzymatic), with ADVIA 1200 equipment – Siemens, Deerfield, Illinois, USA.	
dyslipidaemia	-	Present if low-density lipoprotein cholesterol (LDL-C) levels were ≥130 mg/dL and/or use of lipid-lowering agents. Assessed based on LDL-C in participants' blood samples, collected after a 12-hour fast and analysed using ADVIA 1200 equipment (Siemens, Deerfield, Illinois, USA). LDL-C was estimated by the Friedewald equation when triglycerides (TG) were ≤400 mg/dL and by a homogeneous enzymatic colorimetric method without precipitation when TG levels were >400 mg/dL. The use of lipid-lowering agents, medications for treating dyslipidaemia, was verified through self-report, medical prescriptions, and/or medication packaging presented by the participants.	
hypertension	After the last ELSA telephone interview (or last in-person visit), did a doctor tell you that you have high blood pressure or hypertension?	Self-report of diagnosis and/or by systolic blood pressure (≥140mmHg) and/or diastolic (≥90mmHg) and/or use of antihypertensive medication. Blood pressure (BP) is measured using an automatic oscillometric sphygmomanometer after the participant has rested for five minutes, seated, in a quiet room with controlled temperature. A series of three BP measurements is taken with one-minute intervals between each, recording the average of the last two measurements. Additionally, medical prescriptions or medication packaging presented by participants concerning treatment with antihypertensive drugs in the two weeks preceding the interview were considered: diuretics, beta-blockers, calcium channel blockers, angiotensin-converting enzyme inhibitors, angiotensin II receptor blockers, vasodilators, central and peripheral sympatholytic, as well as a positive response to the question: "Have any of the medications you have taken in the last two weeks been for high blood pressure?"	

Definition of the presence of each morbidity assessed in the Annual Follow-up Interview, during the in-person visit at Wave 2 (2012-2014) and Wave 3 (2017-2019) of the Brazilian Longitudinal Study of Adult Health (ELSA-Brasil)

**Morbidity**	**Assessment during the Annual Follow-up Interview**	**Assessment during the visit to Wave 2**	**Assessment during the visit to Wave 3**
ischaemic heart disease	After the last ELSA telephone interview (or last in-person visit), did a doctor tell you that you had a heart attack or myocardial infarction? Angina, chest pain, or poor circulation in the heart?	-	-
obesity	-	Defined from anthropometric measurements performed in ELSA-Brasil. Body Mass Index (BMI) was estimated by dividing weight (Kg) by the square of height (m²). Participants with a BMI ≥30 Kg/m² were classified as living with obesity.	
renal insufficiency	After the last ELSA telephone interview (or last in-person visit), did a doctor tell you that you have/had renal insufficiency?	-	-
