# Supplementary material for: Racial inequalities in the development of multimorbidity of chronic conditions: results from a Brazilian prospective cohort
Source: Int J Equity Health. 2024 Jun 12;23:120. doi: 10.1186/s12939-024-02201-8 (PMC11170781; doi:10.1186/s12939-024-02201-8)
Supplement: Supplementary file 3 — Supplementary Material 3 [file 12939_2024_2201_MOESM3_ESM.pdf]

Additional File 3

Estimation of time at risk for multimorbidity between Waves 1-2, Waves 2-3, and the entire follow-up period (Waves 1-3) in the Brazilian Longitudinal Study of Adult Health (ELSA-Brasil)

<table>
<thead>
<tr><th>Is there a record of annual follow-up interviews between Waves 1 and 2?</th><th>Is there an in-person visit in Wave 2?</th><th>Is there a record of annual follow-up interviews between Waves 2 and 3?</th><th>Is there an in-person visit in Wave 3?</th><th>Estimation of time at risk between Waves 1 and 2</th><th>Estimation of time at risk between Waves 2 and 3</th><th>Estimation of time at risk throughout the entire period (between Waves 1 and 3)</th></tr>
</thead>
<tbody>
<tr><td>yes</td><td>yes</td><td>yes</td><td>yes</td><td rowspan="4">visit date at Wave 2 minus visit date at Wave 1</td><td rowspan="4">visit date at Wave 3 minus visit date at Wave 2</td><td rowspan="4">visit date at Wave 3 minus visit date at Wave 1</td></tr>
<tr><td>yes</td><td>yes</td><td>no</td><td>yes</td></tr>
<tr><td>no</td><td>yes</td><td>no</td><td>yes</td></tr>
<tr><td>no</td><td>yes</td><td>yes</td><td>yes</td></tr>
<tr><td>yes</td><td>no</td><td>no</td><td>yes</td><td rowspan="4">midpoint date of Wave 2 period minus visit date at Wave 1</td><td rowspan="4">visit date at Wave 3 minus midpoint date of the Wave 2 period</td><td rowspan="4">visit date at Wave 3 minus visit date at Wave 1</td></tr>
<tr><td>yes</td><td>no</td><td>yes</td><td>yes</td></tr>
<tr><td>no</td><td>no</td><td>yes</td><td>yes</td></tr>
<tr><td>no</td><td>no</td><td>no</td><td>yes</td></tr>
<tr><td>yes</td><td>no</td><td>yes</td><td>no</td><td rowspan="2">midpoint date of Wave 2 period minus visit date at Wave 1</td><td rowspan="2">date of the last annual follow-up interview record minus the midpoint date of the Wave 2 period.</td><td rowspan="2">date of the last annual follow-up interview record minus visit date at Wave 1</td></tr>
<tr><td>no</td><td>no</td><td>yes</td><td>no</td></tr>
<tr><td>yes</td><td>yes</td><td>no</td><td>no</td><td rowspan="2">visit date at Wave 2 minus visit date at Wave 1</td><td rowspan="2">zero</td><td rowspan="2">visit date at Wave 2 minus visit date at Wave 1</td></tr>
<tr><td>no</td><td>yes</td><td>no</td><td>no</td></tr>
<tr><td>yes</td><td>yes</td><td>yes</td><td>no</td><td rowspan="2">visit date at Wave 2 minus visit date at Wave 1</td><td rowspan="2">date of the last annual follow-up interview record minus visit date at Wave 2</td><td rowspan="2">date of the last annual follow-up interview record minus visit date at Wave 1</td></tr>
<tr><td>no</td><td>yes</td><td>yes</td><td>no</td></tr>
<tr><td>yes</td><td>no</td><td>no</td><td>no</td><td>date of the last annual follow-up interview record minus visit date at Wave 1</td><td>zero</td><td>date of the last annual follow-up interview record minus visit date at Wave 1</td></tr>
<tr><td>no</td><td>no</td><td>no</td><td>no</td><td>zero</td><td>zero</td><td>zero</td></tr>
</tbody>
</table>
