# Supplementary material for: Racial inequalities in the development of multimorbidity of chronic conditions: results from a Brazilian prospective cohort
Source: Int J Equity Health. 2024 Jun 12;23:120. doi: 10.1186/s12939-024-02201-8 (PMC11170781; doi:10.1186/s12939-024-02201-8)
Supplement: Supplementary file 4 — Supplementary Material 4 [file 12939_2024_2201_MOESM4_ESM.pdf]

Additional File 4

Supplementary Table 1. Adjusted models for potential mediators in the association between racial groups and the development of multimorbidity between Waves 1-2, Waves 2-3, and the entire period (Waves 1-3), Brazilian Longitudinal Study of Adult Health (ELSA-Brasil)

Follow-up	Unadjusted IRR	Adjusted IRR (95% CI)		
	IRR (95% CI)	Model 1	Model 2	Model 3
Between Waves 1 and 2 (*n=4214*)				
Brown (*pardo*)	1.07 (0.93-1.22)	1.19 (1.03-1.37)*	1.15 (0.98-1.33)	1.16 (0.99-1.35)*
Black	1.18 (1.00-1.40)	1.28 (1.07-1.52)**	1.21 (1.01-1.46)*	1.23 (1.02-1.48)*
Between Waves 2 and 3 (*n=3091*)				
Brown (*pardo*)	0.89 (0.74-1.07)	0.99 (0.82-1.20)	1.05 (0.86-1.28)	1.05 (0.85-1.28)
Black	1.04 (0.83-1.31)	1.07 (0.84-1.35)	1.17 (0.91-1.50)	1.18 (0.92-1.51)
Between Waves 1 and 3 (*n=4214*)				
Brown (*pardo*)	1.00 (0.90-1.12)	1.12 (1.00-1.26)	1.11 (0.99-1.26)	1.12 (0.99-1.27)
Black	1.13 (0.99-1.30)	1.20 (1.05-1.38)**	1.20 (1.03-1.39)*	1.21 (1.04-1.41)**

Abbreviations: IRR – Incidence Rate Ratio; 95% CI – 95% Confidence Interval. Notes: the reference category in all models is self-declared white race/skin colour. Model 1 adjusted for age, sex, and ELSA research centre. Model 2 adjusted for Model 1 + educational attainment and monthly *per capita* family income. Model 3 adjusted for Model 2 + smoking and leisure-time physical activity. Significance: *** p-value ≤0.001; ** 0.001 < p-value ≤0.01; * 0.01 < p-value < 0.05.

Supplementary Table 2. Models for the association between racial groups and the development of multimorbidity (list of four morbidities) between Waves 1-2, Waves 2-3, and the entire period (Waves 1-3), Brazilian Longitudinal Study of Adult Health (ELSA-Brasil)

Follow-up	Unadjusted IRR	Adjusted IRR (CI 95%)		
	IRR (CI 95%)	Model 1	Model 2	Model 3
Between Waves 1 and 2 (*n=3982*)				
Brown (*pardo*)	1.06 (0.92-1.22)	1.19 (1.03-1.38)*	1.14 (0.98-1.34)	1.16 (0.99-1.35)
Black	1.19 (1.00-1.41)	1.28 (1.08-1.52)**	1.22 (1.01-1.47)*	1.24 (1.02-1.49)*
Between Waves 2 and 3 (*n=2780*)				
Brown (*pardo*)	0.93 (0.76-1.13)	1.02 (0.83-1.26)	1.05 (0.85-1.30)	1.04 (0.84-1.30)
Black	1.21 (0.95-1.53)	1.21 (0.95-1.54)	1.28 (0.99-1.66)	1.28 (0.99-1.67)
Between Waves 1 and 3 (*n=3613*)				
Brown (*pardo*)	1.03 (0.91-1.16)	1.16 (1.02-1.32)*	1.15 (1.01-1.31)*	1.16 (1.01-1.32)*
Black	1.23 (1.07-1.42)***	1.29 (1.11-1.50)***	1.28 (1.09-1.50)***	1.29 (1.10-1.52)***

Abbreviations: IRR – Incidence Rate Ratio; 95% CI – 95% Confidence Interval. Notes: multimorbidity assessed only by dyslipidaemia, hypertension, obesity, and diabetes. The reference category in all models is self-declared white race/skin colour. Model 1 adjusted for age, sex, and ELSA research centre. Model 2 adjusted for Model 1 + educational attainment and monthly *per capita* family income. Model 3 adjusted for Model 2 + smoking and leisure-time physical activity. The n in parentheses indicates the total number of participants at risk at the beginning of the period, different from that presented in other tables because in this case, only four morbidities are evaluated and only those who attended in-person visits are considered. Significance: *** p-value ≤0.001; ** 0.001 < p-value ≤0.01; * 0.01 < p-value < 0.05.
