# Supplementary material for: Racial inequalities in the development of multimorbidity of chronic conditions: results from a Brazilian prospective cohort
Source: Int J Equity Health. 2024 Jun 12;23:120. doi: 10.1186/s12939-024-02201-8 (PMC11170781; doi:10.1186/s12939-024-02201-8)
Supplement: Supplementary file 5 — Supplementary Material 5 [file 12939_2024_2201_MOESM5_ESM.pdf]

Additional File 5

Prevalence of each morbidity at Wave 1 (2008-2010), Wave 2 (2012-2014), and Wave 3 (2017-2019), participants at risk, new cases, and cumulative incidence for each morbidity, both total and by racial group, Brazilian Longitudinal Study of Adult Health (ELSA-Brasil)

Morbidity	Wave 1		Wave 2		Wave 3		Total number of participants at risk at the beginning of the period (at Wave 1)	New cases of the morbidity during the period (up to Wave 3)	Cumulative incidence per 100 people at risk (95% CI)
	n[(a)]	prevalence at Wave (%)	n[(a)]	prevalence at Wave (%)	n[(a)]	prevalence at Wave (%)			
**Dyslipidaemia**									
Total	4214	864 (20.50)	3960	1.398 (35.30)	3510	1480 (42.17)	3350	1154	34.45 (32.84-36.09)
White	2406	544 (22.61)	2264	826 (36.48)	2010	909 (45.22)	1862	657	35.29 (33.11-37.50)
Brown (*pardo*)	1210	221 (18.26)	1131	381 (33.69)	999	393 (39.34)	989	333	33.67 (30.73-36.71)
Black	598	99 (16.56)	565	191 (33.81)	501	178 (35.53)	499	164	32.87 (28.73-37.18)
p-value[(b)]		*<0.001*		*0.199*		*<0.001*			
**Hypertension**									
Total	4214	354 (8.40)	4214	913 (21.67)	4170	1252 (30.02)	3860	906	23.47 (22.14-24.84)
White	2406	155 (6.44)	2406	436 (18.12)	2379	620 (26.06)	2251	469	20.84 (19.17-22.57)
Brown (*pardo*)	1210	114 (9.42)	1210	297 (24.55)	1199	382 (31.86)	1096	270	24.64 (22.11-27.30)
Black	598	85 (14.21)	598	180 (30.10)	592	250 (42.23)	513	167	32.55 (28.51-36.80)
p-value[(b)]		*<0.001*		*<0.001*		*<0.001*			
**Obesity**									
Total	4214	185 (4.39)	3973	412 (10.37)	3641	474 (13.02)	4029	423	10.50 (9.57-11.49)
White	2406	99 (4.11)	2270	215 (9.47)	2074	240 (11.57)	2307	216	9.36 (8.21-10.63)
Brown (*pardo*)	1210	46 (3.80)	1137	104 (9.15)	1048	138 (13.17)	1164	124	10.65 (8.94-12.57)
Black	598	40 (6.69)	566	93 (16.43)	519	96 (18.50)	558	83	14.88 (12.03-18.10)
p-value[(b)]		*0.011*		*<0.001*		*<0.001*			
**Common nonpsychotic mental disorder**[(c)]									
Total	4214	244 (5.79)		-	3640	466 (12.80)	3970	374	9.42 (8.53-10.37)
White	2406	130 (5.40)		-	2073	238 (11.48)	2276	190	8.35 (7.24-9.56)
Brown (*pardo*)	1210	87 (7.19)		-	1048	155 (14.79)	1123	118	10.51 (8.77-12.45)
Black	598	27 (4.52)		-	519	73 (14.07)	571	66	11.56 (9.05-14.47)
p-value[(b)]		*0.034*				*0.021*			

Abbreviations: 95% CI – 95% Confidence Interval. Notes: (a) n refers to participants with an in-person visit at the Wave (for obesity, dyslipidaemia, and common non-psychotic mental disorders) or at risk at the beginning of the period (for whom recovery of the annual follow-up interview is possible); (b) refers to the X^2 test for differences in proportions between racial groups at the specific wave. (c) Common non-psychotic mental disorders (CMD) were assessed similarly in Waves 1 and 3. In Wave 2, only 6 sections of the 15 applied in Waves 1 and 3 were used, hence there is no score for CMD in Wave 2.

Prevalence of each morbidity at Wave 1 (2008-2010), Wave 2 (2012-2014), and Wave 3 (2017-2019), participants at risk, new cases, and cumulative incidence for each morbidity, both total and by racial group, Brazilian Longitudinal Study of Adult Health (ELSA-Brasil)

Morbidity	Wave 1		Wave 2		Wave 3		Total number of participants at risk at the beginning of the period (at Wave 1)	New cases of the morbidity during the period (up to Wave 3)	Cumulative incidence per 100 people at risk (95% CI)
	n[(a)]	prevalence at Wave (%)	n[(a)]	prevalence at Wave (%)	n[(a)]	prevalence at Wave (%)			
**Diabetes**									
Total	4214	95 (2.25)	4214	278 (6.60)	4170	422 (10.12)	4119	329	7.99 (7.18-8.86)
White	2406	41 (1.70)	2406	135 (5.61)	2379	210 (8.83)	2365	170	7.19 (6.18-8.30)
Brown (*pardo*)	1210	34 (2.81)	1210	89 (7.36)	1199	131 (10.93)	1176	97	8.25 (6.74-9.97)
Black	598	20 (3.34)	598	54 (9.03)	592	81 (13.68)	578	62	10.73 (8.32-13.54)
p-value[(b)]		*0.016*		*0.005*		*0.001*			
**Cancer**									
Total	4214	40 (0.95)	4214	78 (1.85)	4170	225 (5.40)	4174	188	4.50 (3.90-5.18)
White	2406	29 (1.21)	2406	56 (2.33)	2379	164 (6.89)	2377	138	5.81 (4.89-6.82)
Brown (*pardo*)	1210	8 (0.66)	1210	18 (1.49)	1199	45 (3.75)	1202	37	3.08 (2.18-4.22)
Black	598	3 (0.50)	598	4 (0.67)	592	16 (2.70)	595	13	2.19 (1.17-3.71)
p-value[(b)]		*0.134*		*0.014*		*<0.001*			
**Ischaemic heart disease**									
Total	4214	7 (0.17)	4214	27 (0.64)	4170	70 (1.68)	4207	64	1.52 (1.17-1.94)
White	2406	6 (0.25)	2406	18 (0.75)	2379	43 (1.81)	2400	38	1.58 (1.12-2.17)
Brown (*pardo*)	1210	1 (0.08)	1210	6 (0.50)	1199	17 (1.42)	1209	16	1.32 (0.76-2.14)
Black	598	0 (0.00)	598	3 (0.50)	592	10 (1.69)	598	10	1.67 (0.81-3.05)
p-value[(b)]		*0.285*		*0.602*		*0.693*			
**Cerebrovascular accident**									
Total	4214	3 (0.07)	4214	11 (0.26)	4170	19 (0.46)	4211	16	0.38 (0.22-0.62)
White	2406	2 (0.08)	2406	6 (0.25)	2379	9 (0.38)	2404	7	0.29 (0.12-0.60)
Brown (*pardo*)	1210	1 (0.08)	1210	4 (0.33)	1199	8 (0.67)	1209	7	0.58 (0.23-1.19)
Black	598	0 (0.00)	598	1 (0.17)	592	2 (0.34)	598	2	0.33 (0.04-1.21)
p-value[(b)]		*0.780*		*0.803*		*0.432*			

Abbreviations: 95% CI – 95% Confidence Interval. Notes: (a) n refers to participants with an in-person visit at the Wave (for obesity, dyslipidaemia, and common non-psychotic mental disorders) or at risk at the beginning of the period (for whom recovery of the annual follow-up interview is possible); (b) refers to the X^2 test for differences in proportions between racial groups at the specific wave. (c) Common non-psychotic mental disorders (CMD) were assessed similarly in Waves 1 and 3. In Wave 2, only 6 sections of the 15 applied in Waves 1 and 3 were used, hence there is no score for CMD in Wave 2.

Prevalence of each morbidity at Wave 1 (2008-2010), Wave 2 (2012-2014), and Wave 3 (2017-2019), participants at risk, new cases, and cumulative incidence for each morbidity, both total and by racial group, Brazilian Longitudinal Study of Adult Health (ELSA-Brasil)

**Morbidity**	**Wave 1**		**Wave 2**		**Wave 3**		**Total number of participants at risk at the beginning of the period (at Wave 1)**	**New cases of the morbidity during the period (up to Wave 3)**	**Cumulative incidence per 100 people at risk (95% CI)**
	**n[(a)]**	**prevalence at Wave (%)**	**n[(a)]**	**prevalence at Wave (%)**	**n[(a)]**	**prevalence at Wave (%)**			
**Cardiac insufficiency**									
Total	4214	4 (0.09)	4214	5 (0.12)	4170	17(0.41)	4210	13	0.31 (0.17-0.53)
White	2406	2 (0.08)	2406	2 (0.08)	2379	9 (0.38)	2404	7	0.29 (0.12-0.60)
Brown (*pardo*)	1210	1 (0.08)	1210	1 (0.08)	1199	4 (0.33)	1209	3	0.25 (0.05-0.72)
Black	598	1 (0.17)	598	2 (0.33)	592	4 (0.68)	597	3	0.50 (0.10-1.46)
p-value[(b)]		*0.825*		*0.254*		*0.533*			
**Renal insufficiency**									
Total	4214	209 (4.96)	4214	211 (5.01)	4170	215 (5.16)	4005	9	0.23 (0.10-0.43)
White	2406	134 (5.57)	2406	134 (5.57)	2379	136 (5.72)	2272	2	0.09 (0.01-0.32)
Brown (*pardo*)	1210	53 (4.38)	1210	54 (4.46)	1199	57 (4.75)	1157	6	0.52 (0.19-1.13)
Black	598	22 (3.68)	598	23 (3.85)	592	22 (3.72)	576	1	0.17 (0.004-0.96)
p-value[(b)]		*0.089*		*0.132*		*0.109*			

Abbreviations: 95% CI – 95% Confidence Interval. Notes: (a) n refers to participants with an in-person visit at the Wave (for obesity, dyslipidaemia, and common non-psychotic mental disorders) or at risk at the beginning of the period (for whom recovery of the annual follow-up interview is possible); (b) refers to the X^2 test for differences in proportions between racial groups at the specific wave. (c) Common non-psychotic mental disorders (CMD) were assessed similarly in Waves 1 and 3. In Wave 2, only 6 sections of the 15 applied in Waves 1 and 3 were used, hence there is no score for CMD in Wave 2.
